# AFFECTIVE NEUROPSYCHIATRIC SYMPTOMS MAY BE EARLY SIGNS OF ALZHEIMER'S DISEASE IN NON-DEMENTED OLDER ADULTS

**DOI:** 10.1093/geroni/igz038.3520

**Published:** 2019-11-08

**Authors:** Jung Y Jang, Daniel A Nation

**Affiliations:** 1 University of Southern California, Los Angeles, California, United States; 2 University of California, Irvine, Irvine, California, United States

## Abstract

The current study sought to investigate the association between affective neuropsychiatric symptoms (aNPS: depression, anxiety, apathy, irritability), Alzheimer’s disease (AD) cerebrospinal fluid (CSF) biomarkers profiles, and the risk of progression to dementia in non-demented older adults. Participants consisted of 763 individuals with normal cognition (CN) (mean age = 73.73 ± 6.68) and 617 with mild cognitive impairment (MCI) (mean age = 73.19 ± 7.40) at baseline, who were enrolled in the Alzheimer’s Disease Neuroimaging Initiative (ADNI). Latent class analyses (LCA) identified three subgroups of older adults within CN and MCI, respectively, showing distinct patterns of the neuropsychiatric inventory (NPI) domains. Results indicated that the subgroup with higher probabilities of aNPS had elevated risk of progression to dementia (HR = 3.18, 95% CI [1.70, 5.94] in CN, HR = 1.79, 95% CI [1.01, 3.16] in MCI), adjusting for age, sex, and Apolipoprotein E e4 (APOE4) carrier status. Subgroups did not differ in their profiles of AD CSF biomarkers. Findings suggest that aNPS might be symptoms of secondary disease processes in the brain, lowering the threshold for AD pathophysiology to manifest clinically in CN and MCI. The current study highlights the importance of assessment and interventions for emotional and behavioral symptoms in non-demented older adults.

